# Application of simplified MLST scheme for direct typing of clinical samples from human leptospirosis cases in a tertiary hospital in the Philippines

**DOI:** 10.1371/journal.pone.0258891

**Published:** 2021-10-20

**Authors:** Marjo V. Mendoza, Windell L. Rivera

**Affiliations:** 1 Pathogen-Host-Environment Interactions Research Laboratory, Institute of Biology, College of Science, University of the Philippines Diliman, Quezon City, Philippines; 2 Natural Sciences Research Institute, University of the Philippines Diliman, Quezon City, Philippines; Bharathidasan University, INDIA

## Abstract

Despite the major threat of leptospirosis to public health in the Philippines, its epidemiologic data remain scarce. Multilocus sequence typing (MLST) is a method often used for identification of circulating *Leptospira* species and disease surveillance. Unfortunately, molecular typing of *Leptospira* isolates is not routinely done in most hospital settings. A simplified MLST scheme targeting three loci (*adk*, *lipL41*, *mreA*) was performed for rapid direct typing of *Leptospira* in clinical specimens. Blood samples from suspected or clinically diagnosed cases (n = 50) were initially screened via polymerase chain reaction (PCR) targeting 23S rRNA, 16S rRNA (*rrs2*), and *lipL32* genes. From the nine positives, seven had interpretable data from MLST. Allelic profiles identified *L*. *interrogans* in all positive samples. Six were assigned to ST12 of serovar Manilae (serogroup Pyrogenes) while one sample cannot be clearly differentiated between two serovars/serogroups, Bataviae/Losbanos (serogroup Bataviae) or Australis (serogroup Australis), indicating possibility of a new ST. Phylogenetic analysis confirmed that the application of simplified MLST scheme produces consistent results with the seven-loci genetic profile of published *Leptospira* MLST schemes. Reduced scheme addressed the challenges often encountered in the amplification of full MLST genetic profile of *Leptospira*. The approach is a potential alternative to serological tests for rapid typing of clinical specimens and can also aid in investigations on disease epidemiology specifically to monitor occurrence, pathogen transmission, host specificity and susceptibility, and other factors that could lead to potential outbreaks.

## Introduction

Leptospirosis is an emerging zoonotic disease with significant health and economic impact worldwide [1]. Although it is considered endemic in tropical countries, increasing prevalence and widespread distribution have been reported in recent years particularly in developing countries in South and Southeast Asia, Western Pacific, Central and South America, and East African regions [2]. Disease outbreaks are attributed to both ecological and environmental factors such as climate change, urbanization, exposure to contaminated water and infected animals, and unsanitary living conditions as a result of population growth [3, 4]. However, the actual global incidence of leptospirosis is still unknown. Lack of data is possibly due to misdiagnosis and underreporting of cases because of inadequate diagnostic laboratory services in areas with high disease burden [5].

Past report on leptospirosis overall morbidity and mortality estimated 1.03 million cases and 58,900 deaths annually. Male population ranging from 20 to 49 years of age was recorded to have the highest case numbers and mortality rate [6]. In the Philippines, leptospirosis has been included in the disease surveillance system of the Department of Health (https://www.doh.gov.ph/statistics) because of increasing incidence in the past five years. The highest number of cases recorded was in 2018 with 5,116 cases and 505 deaths nationwide, which is 71% higher than the 2017 cases (3,067). Surge of cases was from areas with most frequent typhoon occurrence specifically in the National Capital Region (NCR) and Regions I, III, IV-A and VI [7].

Characterization of pathogen’s genetic diversity is a major key to understanding the epidemiology of infectious diseases [8, 9]. Molecular typing of pathogens has made substantial impact on disease diagnosis and surveillance. However, majority of the conventional methods are laborious, time-consuming, and often produce inconsistent results [10]. Technological advancements in molecular biology provided deeper insight into the epidemiology of infectious diseases. In fact, molecular studies continuously generate information on genetic profiles of pathogens, infection prevalence and geographic distribution, complex transmission pathways, risk factors, and disease pathogenesis [9]. The advent of next-generation sequencing (NGS) offered a more accurate and comprehensive analysis of microbial diversity at the genomic level. Unfortunately, this tool presented major challenges in terms of accessibility to sequencing facilities and data analysis [11]. Multilocus sequence typing (MLST) provided a flexible way of characterizing pathogens by targeting at most seven well-conserved loci in the genome, thus, it has been widely used for molecular typing and other epidemiologic investigations [12]. For leptospirosis, MLST is currently explored as possible alternative for classification of pathogenic *Leptospira* species. It has shown considerable advantages by providing consistent and reproducible results compared to classical typing methods such as microscopic agglutination test (MAT) [13–15].

Three schemes with high discriminatory power are available in the *Leptospira* PubMLST database targeting six to seven loci [16–18]. The problem with standard MLST schemes is the difficulty in generating full genetic profile directly from clinical samples [13, 19, 20]. Because of the unreliability of PCR amplification of these established markers in this type of samples, optimization of an alternative reduced MLST scheme with comparable typing efficiency and discriminatory power to a full MLST scheme may be necessary for rapid identification of *Leptospira* clinical isolates. Implementation of such tool would be advantageous for routine use since it would reduce processing time and costs, allowing characterization of outbreak transmission pathways and clusters, as well as identification of species infecting multiple hosts. In this study, previously described reduced MLST scheme targeting three loci [21] was adapted to classify *Leptospira* species in human clinical samples obtained from a tertiary hospital in the NCR, the region with highest recorded cases in the Philippines in 2018. The goal of this study was to analyze genetic diversity of *Leptospira* species causing human infection in the Philippines and to evaluate the accuracy of the simplified MLST scheme in direct clinical sample typing. To our knowledge, this is the first report to perform MLST in Philippine clinical isolates. However, the limited sample size attributed to the difficulty in accessing *Leptospira* clinical samples in Philippine hospitals means that the results may not necessarily reflect the variability of *Leptospira* sp. in the Philippines. Moreover, a larger number of samples is needed to validate the applicability of the reduced scheme. Still, results of this study may provide additional information on the etiology of the disease. Such information is crucial in conducting risk assessment and implementation of control strategies to mitigate the spread of infection.

## Materials and methods

### Ethics statement

Prior to conduct of this study, ethical approval was obtained from the Technical/Ethical Review Board of the Ospital ng Maynila Medical Center (OMMC), Philippines. No new samples were collected from patients, instead, anonymized residual blood samples previously submitted for diagnostic tests were used.

### Clinical samples

The sample size is dependent on the number of clinical samples available at the hospital during the collection period. Blood samples (n = 50) from patients suspected or clinically diagnosed with leptospirosis in 2017–2018 at the OMMC were obtained. Specifically, samples from patients who upon admission presented with acute febrile illness, other symptoms associated with leptospirosis, and had high risk exposure to contaminated environment or infected animals were included in the study.

### Detection and identification of *Leptospira* spp.

DNA was isolated from 200 μl whole blood using Chelex® 100 (Bio-Rad) and PureLink™ Genomic DNA Mini Kit (Invitrogen). *Leptospira* DNA was detected by polymerase chain reaction (PCR) targeting 23S rRNA [22] and 16S rRNA (*rrs2*) genes, which identify all known species of *Leptospira*, and *lipL32* gene [18], which is present in pathogenic *Leptospira* species only. Amplifications were done using GoTaq® Master Mix (Promega) and was carried out in T100™ Thermal Cycler (Bio-Rad). Cycling conditions consisted of the following: initial denaturation at 95°C for 5 min, followed by 30 cycles of denaturation at 94°C for 30 sec, annealing at 54°C for 30 sec, extension at 72°C for 1 min for the 23S and *rrs2* gene targets. Same conditions were used for *lipL32* gene amplification except for the annealing temperature set at 56°C. Final extension was at 72°C for 10 min. Amplicons were confirmed by electrophoresis on 1.5% agarose gel using SYBR® Safe DNA Gel Stain (Invitrogen). Samples were tested at least twice for each gene target. Amplicons of *rrs2* and *lipL32* genes were subsequently sent to Macrogen, Inc. (Seoul, Korea) for sequencing to confirm the identity of *Leptospira* in positive samples. All sequences produced in the present study were submitted to the NCBI GenBank database (S1 Table).

### MLST schemes

MLST was performed on *Leptospira* positive samples using the previously described simplified scheme targeting three loci (*adk*, *lipL41*, *mreA*) [21] and published schemes 2 (*adk-glmU-icdA-lipL32-lipL41-mreA-pntA*) and 3 (*adk-icdA-lipL32-lipL41-rrs2-secY*) in the PubMLST database (https://pubmlst.org/leptospira/) [23]. MLSTest software v 1.0.1.23 [24] was used to calculate the typing efficiency and discriminatory power of each locus and MLST schemes. PCR amplifications were done in a volume of 25 μl containing 13 μl GoTaq® Master Mix (Promega), 0.5 μl (10 μM) each of forward and reverse primers, 9 μl nuclease-free water, and 2 μl DNA template. Cycling conditions consisted of an initial denaturation at 95°C for 5 min, followed by 40 cycles of denaturation at 94°C for 30 sec, annealing between 48–56°C for 30 sec, extension at 72°C for 1 min, and final extension at 72°C for 7 min. Primer sequences and annealing temperatures used in this study are provided in S2 Table. Amplicons were also sent to Macrogen, Inc. for sequencing. Allelic profiles and sequence types (STs) were assigned in the PubMLST database [23].

### Phylogenetic analysis

Phylogenetic trees were constructed based on the single gene (*rrs2*), simplified three MLST loci (*adk-lipL41-mreA*) and seven MLST loci (*adk-lipL41-mreA-lipL32-pntA-rrs2-secY*) genetic profiles. Reference sequences for phylogenetic analyses were exported from *Leptospira* PubMLST database including deposited sequences of Philippine isolates (S3 Table). Reference isolates from 11 serogroups of *L*. *interrogans* were represented in the phylogenetic tree construction and *L*. *kirschneri* sequences were used as outgroup. Multiple sequences of *adk* (430 bp), *lipL41* (492 bp), *mreA* (435 bp), *lipL32* (450 bp), *pntA* (525 bp), *rrs2* (450 bp), and *secY* (461 bp) were aligned and trimmed separately using the ClustalW algorithm in BioEdit v 7.2.5 [24]. Sequences of the three loci (1,357 bp) and seven loci (3,243 bp) were concatenated in DAMBE v 5.2.5 [25] and the concordance between concatenated sequences were checked by performing the Incongruence Length Test (ILT) in PAUP* v 4.0b10 program [26]. Oversaturation was also assessed by Xia test in DAMBE v 5.2.5. Optimal DNA substitution models for *rrs2* and concatenated datasets were determined based on Bayesian Information Criterion (BIC) in jModelTest v 2.1.10 software [27]. Maximum-Likelihood (ML) and Neighbor-Joining (NJ) tree construction methods were performed with 1,000 bootstrap replicates in PhyML 3.1 program [28] and PAUP* v 4.0b10 program, respectively. Trees were based on the parameters of the optimal models of DNA substitution identified, K80 (*rrs2*), HKY+G (three loci), and TPM3uf+I+G (seven loci). NJ and ML trees were drawn in TreeExplorer v 2.12 software program [29].

## Results

### *Leptospira* detection and identification in human clinical samples

PCR-based detection of *Leptospira* revealed two positives for 23S rRNA and nine (18%) positives for both *rrs2* and *lipL32* from the 50 human clinical specimens tested (Table 1). Only POM01 and POM07 were positive for all diagnostic gene targets. Results were not validated by MAT and culture method because of unavailability of tests in the hospital laboratory. Sequencing of *rrs2* and *lipL32* genes identified all isolates as *Leptospira interrogans*. Serovar or serogroup was not determined.

**Table 1:** PCR-positive samples for *Leptospira* detection and MLST loci.

Sample code	*23S* ^*a*^	*rrs2* ^*a*^ ^,^ ^*b*^	*lipL32* ^*a*^ ^,^ ^*b*^	*adk* ^*b*^	*glmU* ^*b*^	*icdA* ^*b*^	*lipL41* ^*b*^	*mreA* ^*b*^	*pntA* ^*b*^	*secY* ^*b*^	Species Identification (*rrs2/lipL32*)
POM01	+	+	+	+	-	+	+	+	+	+	*L*. *interrogans*
POM03	-	+	+	-	-	-	-	-	-	-	*L*. *interrogans*
POM07	+	+	+	+	-	+	+	+	+	+	*L*. *interrogans*
POM08	-	+	+	-	-	-	-	-	-	-	*L*. *interrogans*
POM18	-	+	+	+	-	+	+	+	+	+	*L*. *interrogans*
POM19	-	+	+	+	-	+	+	+	+	+	*L*. *interrogans*
POM20	-	+	+	+	-	+	+	+	+	+	*L*. *interrogans*
POM23	-	+	+	+	-	+	+	+	+	+	*L*. *interrogans*
POM37	-	+	+	+	-	+	+	+	+	+	*L*. *interrogans*

± indicates positive or negative amplification for each locus.

^*a*^gene targets for PCR-based *Leptospira* detection.

^*b*^loci included in *Leptospira* MLST.

### Reduced scheme optimization

Direct typing of *Leptospira* in clinical samples is very difficult to perform. Some genes are not easily amplified, hence, the need for a reduced MLST scheme applicable for rapid identification of serovars in clinical setting. By implementing MLSTest, all possible combinations from 1 to 7 loci were assessed on two sets of samples. The first set includes Philippine isolates only and the second includes both Philippine and reference isolates. Because of the low number of isolates in the Philippine sample set, discriminatory power (DP) of each locus was low ranging from 0.25 to 0.893 (Table 2). Selection of optimal scheme revealed same maximum number of 5 STs for each loci combination, thus a reduced MLST scheme was not identified for this sample set. The addition of reference isolates showed increased DP for each single locus ranging from 0.352 (*lipL32*) to 0.881 (*secY*). High DP was observed in *adk*, *lipL41*, *mreA*, *pntA*, and *secY*. Typing efficiency (TE) values were 0.18 (*adk*) to 1.333 (*rrs2*). Optimum number of possible loci that can identify maximum ST were determined through scheme optimization (Table 3). From the 127 combinations analyzed, *adk* and *pntA* 2-loci combination found a maximum of 16 STs. However, the scheme was deemed unsuitable for routine use due to difficulty in PCR optimization of *pntA* requiring multiple amplifications, thus this target was excluded. Focusing on the *adk-lipL41-mreA* and *adk-mreA-secY* loci combinations, schemes revealed TE of 0.263 and 0.213, respectively, and same DP values of 0.963 (0.903–1) with 95% confidence interval. Maximum of 16 STs were also obtained in both schemes similar to the 7-loci scheme, which had 0.963 DP and 0.256 TE. Although there is minor difference in the results of both schemes, *adk-lipL41-mreA* loci combination has better TE. Also, *adk*, *lipL41*, and *mreA* are among the markers with highest number of alleles reported in the PubMLST database (scheme 2) to date, with 100, 92, and 80 alleles respectively, hence, the scheme was selected for the reduced MLST.

**Table 2:** Typing efficiency and discriminatory power of the markers analyzed for the reduced MLST scheme selection.

	Philippine isolates				Philippine and Reference isolates			
Markers	Number of alleles	Number of Polymorphisms	Typing efficiency	Discriminatory Power	Number of alleles	Number of Polymorphisms	Typing efficiency	Discriminatory Power
*adk*	5	5	1	0.893	9	50	0.18	0.848
*lipL32*	1	0	inf	0.25	4	6	0.667	0.352
*lipL41*	1	0	inf	0.25	8	25	0.32	0.8
*mreA*	2	3	0.667	0.464	9	35	0.257	0.867
*pntA*	1	0	inf	0.25	9	41	0.22	0.833
*rrs2*	2	1	2	0.607	4	3	1.333	0.548
*secY*	4	8	0.5	0.786	11	51	0.216	0.881
3-loci*					16	110	0.263	0.963
3-loci**					16	136	0.213	0.963
7-loci					16	211	0.256	0.963

*adk-lipL41-mreA.

**adk-mreA-secY.

**Table 3:** Optimum number of loci combination based on MLSTest.

Number of Loci (number of combinations)	Maximum number of STs found	ST/Strain ratio
1 (7)	11	0.55
2 (21)^a^	16	0.8
3 (35)^b^	16	0.8
4 (35)	16	0.8
5 (21)	16	0.8
6 (7)	16	0.8
7 (1)	16	0.8

^a^ optimum loci combination: *adk-pntA*.

^b^ optimum loci combinations: *adk-lipL41-mreA*, *adk-lipL41-pntA*, *adk-mreA-lipL32*, *adk-mreA-pntA*, *adk-mreA-secY*, *adk- lipL32-pntA*, *adk-pntA-rrs2*, *adk-pntA-secY*.

### Comparison of simplified and original MLST schemes

Three MLST schemes were performed to confirm the ST of *Leptospira* in positive clinical samples. Nine loci based from MLST schemes 2 and 3 in the *Leptospira* PubMLST database were used. Results showed seven samples were positive for eight loci (*rrs2*, *lipL32*, *adk*, *icdA*, *lipL41*, *mreA*, *pntA*, *secY*) (Table 1). *glmU* could not be amplified in any sample. POM03 and POM08 were removed from analysis since they yielded positive results only for *rrs2* and *lipL32* genes. Three loci of the simplified MLST scheme provided interpretable data from seven clinical samples. All sequences were used for genotyping except for *icdA*, which had ambiguous sequences in all samples.

Based on the sequences, exact allele matches were identified for the *lipL32*, *lipL41*, *mreA*, and *pntA* loci. Partial matches for *adk*, *rrs2*, and *secY*, on the other hand, were noted in some samples due to shorter sequence length amplified. Allelic profiles of samples from simplified and original MLST schemes are reported in S4 Table. Full allelic profiles from the simplified scheme (*adk-lipL41-mreA*) identified scheme 2 ST12 in six samples (POM01, POM07, POM18, POM19, POM20, POM23). POM37 probable assignment was scheme 2 ST24 or ST25, which had the nearest profile match. Exact ST could not be determined for POM37 because *lipL41* was found to match allele 8 and not allele 19 or 20 of ST24 and ST25, respectively. Same STs were identified based on original MLST scheme 2 partial allelic profiles for five loci (*adk-lipL32-lipL41-mreA-pntA*). *pntA* of POM37 corresponding to allele 6 did not match with allele 14 of ST24 and ST25. MLST scheme 3 assigned six samples to ST9. POM37 nearest profile match were ST44 and ST49.

Presumptive serovar and serogroup were identified based on the STs deposited in the PubMLST database (Table 4). Six samples linked to scheme 2 ST12 or scheme 3 ST9 were identified as *L*. *interrogans* serovar Manilae (serogroup Pyrogenes). POM37 nearest ST matches corresponded to *L*. *interrogans* with Bataviae/Losbanos (serogroup Bataviae) or Australis (serogroup Australis) serovars. However, it should be noted that POM37 may be assigned to a new ST depending on the sequence of missing alleles. Comparison of deposited isolates in *Leptospira* PubMLST seen in S5 Table showed six clinical samples and five reference isolates sharing identical sequence type, scheme 2 ST12 or scheme 3 ST9. Among these reference isolates was LT398 which was isolated from a rat host in the Philippines in 1957. Other reference isolates, UP-MMC-NIID HP and UP-MMC-NIID LP, were reported from Japan, both collected from a mouse. No information was provided for Manilae str. K56 and Manilae str. UP-OM. POM37 shared same STs with six reference isolates, Swart and LT101-69 (scheme 3 ST44) and Ballico, SRR507773, Australis str. 200703203 and SU5 (scheme 3 ST49). Isolate LT101-69 was also from a rat captured in the Philippines.

**Table 4:** Presumptive STs and serogroup of *Leptospira* in clinical samples based on simplified and published MLST schemes.

Sample code	Presumptive ST			Species	Presumptive Serovar^a^	Presumptive Serogroup^b^
	Simplified MLST	MLST Scheme 2	MLST Scheme 3			
POM-01	12	12*	9*	*L*. *interrogans*	Manilae	Pyrogenes
POM-03	NA	NA	NA	*L*. *interrogans*	NA	NA
POM-07	12	12*	9*	*L*. *interrogans*	Manilae	Pyrogenes
POM-08	NA	NA	NA	*L*. *interrogans*	NA	NA
POM-18	12	12*	9*	*L*. *interrogans*	Manilae	Pyrogenes
POM-19	12	12*	9*	*L*. *interrogans*	Manilae	Pyrogenes
POM-20	12	12*	9*	*L*. *interrogans*	Manilae	Pyrogenes
POM-23	12	12*	9*	*L*. *interrogans*	Manilae	Pyrogenes
POM-37	24, 25	24*, 25*	44*	*L*. *interrogans L*. *interrogans*	Bataviae Losbanos Australis	Bataviae Australis
			49*			

Sequence type (ST) assignment of each sample was based on original MLST schemes 2 and 3. Probable ST for simplified scheme was based on scheme 2 allele assignments.

NA—not assigned due to limited number of genes amplified.

*Samples with partial allelic profile in which nearest ST match was inferred from available loci only.

^a, b^Presumptive serovar and serogroup were based on ST match in *Leptospira* PubMLST database (https://pubmlst.org/leptospira/).

### Phylogeny of *Leptospira* based on *rrs2* and MLST loci

Phylogeny was inferred from single gene (*rrs2*), simplified three MLST loci (*adk-lipL41-mreA*) and seven available MLST loci (*adk-lipL41-mreA-lipL32-pntA-rrs2-secY*) in the study. Reference sequences from 13 isolates, 12 *L*. *interrogans* and one *L*. *kirschneri*, were obtained from *Leptospira* PubMLST. *L kirschneri* isolate Wumalasena was used as an outgroup. Of the 885 *L*. *interrogans* isolates currently deposited in the database, five were from the Philippines, however, only three (LT398, LT101-69, C3) have complete genetic profile. The other two Philippine reference isolates were excluded from analysis. Eleven of the 16 serogroups of *L*. *interrogans* in the database were represented in the phylogeny. Isolates from serogroup Javanica, Lyme, Mini, Ranarum, and Sehgali have incomplete genetic profile, thus cannot be included in the analysis.

Alignment of *rrs2* (450 nt) confirmed high sequence homology within *L*. *interrogans* species, hence phylogenetic tree constructed based on ML and NJ methods poorly resolved serovar subgroups (S1 Fig). All seven samples clustered with nine reference isolates including those from the Philippines with >60% bootstrap support. POM01 and POM19 formed a subgroup within the clade. It was also noted that *rrs2* assigned three *L*. *interrogans* (serogroup Hebdomadis, Pomona, Sejroe) and *L*. *kirschneri* in the same clade.

**Fig 1:**
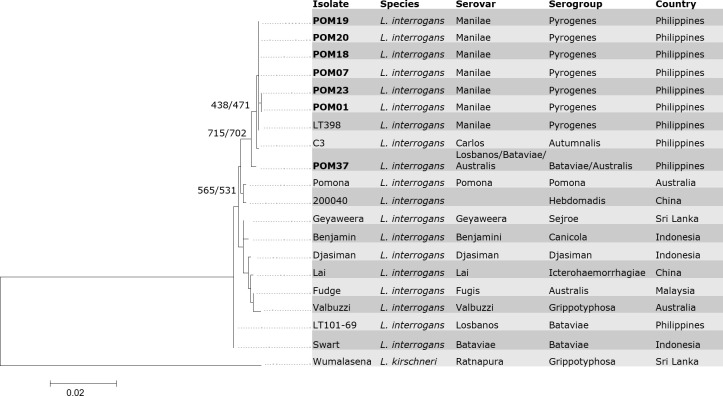
Maximum Likelihood tree of *Leptospira* inferred from three MLST loci (*adk-lipL41-mreA*) gene sequence alignment (1,357 nt length) based on HKY+G DNA substitution model. Clinical samples (n = 7) are in bold and their corresponding species, serovar, and serogroup assignments are listed. Reference *L*. *interrogans* sequences represent 11 serogroups reported in the database (https://pubmlst.org/leptospira/). The tree was rooted with the outgroup, *L*. *kirschneri*. Bootstrap values with 1,000 replicates inferred from ML and NJ are shown in the nodes. Scale bar represents 0.02 substitution per site. Corresponding GenBank accession numbers of isolates are provided in S1 Table. Isolate ID of reference sequences from *Leptospira* PubMLST database are reported in S3 Table.

Phylogeny based on the simplified and original MLST schemes provided increased phylogenetic signals and better resolution. Incongruence test using 15 parsimony-informative characters out of the 1,357 total nucleotides of the *adk-lipL41-mreA* sequences confirmed that the genes can be concatenated (0.03 *p* value). Same ILT results were obtained in the seven loci concatenated sequences (3,243 nt) with 0.02 *p* value. Saturation analyses revealed significantly low level of saturation (Iss < Iss.c) in each concatenated sequence dataset. NJ and ML trees constructed based on three loci concatenated sequences of (Fig 1) are similar, but not identical, with the seven loci tree (S2 Fig). Nine out of 10 Philippine isolates including two reference isolates, LT398 and C3, formed a single clade with >70% bootstrap support. LT101-69 reference Philippine isolate did not cluster with other isolates. Although supported by weak bootstrap (>50%), six samples (POM01, POM07, POM18, POM19, POM20, POM23) clustered in a single clade together with LT398 confirming that they share same sequence type, scheme 2 ST12. POM37 remained separated from other Philippine samples, neither did it cluster with isolate Swart nor LT101-69 of scheme 2 ST24 and ST25, its nearest ST matches. POM37 instead appeared to be more closely related to C3, a Philippine isolate collected from a toad. Interestingly, C3 allelic profile (13-14-9) did not match with POM37.

## Discussion

Molecular techniques for microbial typing have been developed to facilitate epidemiologic investigations [10]. MLST has become a standard for this purpose because of its discriminatory power, ease of application and interpretation of results, and availability of a comprehensive central database in which all existing genetic profiles can be easily accessed for comparison. Such tool presents an advantage in clinical setting, particularly in diagnosis of microbial infections [30]. In this study, a simplified MLST scheme was performed to classify *Leptospira* species directly from human clinical specimens. Whole blood samples initially obtained from probable leptospirosis cases were screened via PCR targeting three genes. Only 14% were confirmed positive by *rrs2* and *lipL32* gene amplifications. 23S rRNA gene had lower sensitivity with only two positive samples detected. The result is in contrast with previous study wherein 23S rRNA based detection exhibited higher sensitivity compared to *rrs2* even for archived tissues [31]. *rrs2* and *lipL32* are widely used as diagnostic markers in most assays for *Leptospira* detection. Although some studies produced contradicting results, depending on the length of amplified fragment, both genes have demonstrated relatively high sensitivity and specificity in screening clinical specimens [32–34]. The negative results do not necessarily imply that *Leptospira* is not the infective agent. Poor sample quality, PCR primer design, low leptospiremia due to late clinical presentation, and empiric antibiotic treatment may account for these findings [34, 35]. Note that clinical samples in this study were not screened by culture or MAT, the gold standards for leptospirosis detection. Diagnosis was only done based on laboratory findings and clinical presentation of patients such as acute febrile illness, headache, myalgia, and prostration after exposure to contaminated environment or infected animals. This practice present challenges and often result to misdiagnosis possibly because of the nonspecific manifestations and the range of leptospirosis symptoms, hence the need for a more sensitive and faster diagnostic tool [1, 36].

*Leptospira* typing based on simplified and original MLST schemes 2 and 3 revealed partial *Leptospira* allelic profiles in clinical samples. MLST scheme 1 was not performed because the simplified scheme was based on published scheme 2 and its genetic profile is quite different from the other original schemes [23]. No new alleles were identified in the study. Sequence type assignment revealed *L*. *interrogans* infection in all positive samples, six were classified as serovar Manilae. In the Philippines, pathogenic species that have been isolated from both humans and animals are *L*. *interrogans*, *L*. *borgpetersenii*, and *L*. *kirschneri*. *L*. *interrogans* causes majority of human infections in the country and the most prevalent serovars or serogroup are Pyrogenes, Bataviae, Grippotyphosa, Manilae, Losbanos, Tarassovi, and Poi [37–40]. Manilae sequence type was detected in nearly all samples based on MLST result which was further confirmed through phylogenetic analysis. Interestingly, the same sequence type was already isolated 60 years ago in the country from a rat host. This could mean that this is the most predominant circulating serovar in the country for the past 60 years. POM37 serovar assignment was not conclusive since no exact ST match was found. MLST linked it to Bataviae, Losbanos, and Australis serovars, however, based on phylogeny, its genetic profile is more likely related to C3 (serogroup Australis), also a Philippine isolate from a toad host. Sequencing of additional loci using a different scheme may provide more information on this isolate or a new sequence type can be assigned to it.

MLST simplified and original schemes yielded similar results despite the incomplete allelic profiles in the original MLST schemes. Previous study has demonstrated the reliability of *adk-lipL41-mreA* loci combination in accurately classifying *Leptospira* in clinical specimens despite reduced profile. The scheme retained the discriminatory power of original MLST scheme with highest ST/strain ratio compared to other loci combination [21]. The use of housekeeping and non-housekeeping genes in the reduced scheme may account for this since both have also produced robust phylogeny with better resolution in other species [30]. Housekeeping genes *adk* and *mreA* have high nucleotide diversity and more clonal complexes despite slow evolution rate in *Leptospira* [17]. *lipL41*, a non-housekeeping gene which encodes for a leptospiral outer membrane lipoprotein for cellular adhesion, was shown to have higher rate of variability. Antigenic diversity among *Leptospira* serovars have been attributed to structural and genetic variations of membrane proteins. Hence, the inclusion of *lipL41* present an advantage by allowing intraspecies discrimination in pathogenic leptospires [41]. The reliability of genetic markers is important in species identification and assessment of phylogenetic relationships. Housekeeping genes are good markers because of their slow evolution rate, thus retaining the relationship between strains and providing accurate estimation of phylogeny. Non-housekeeping genes, on the other hand, may be fast-evolving but they provide more phylogenetic signals which permit assessment of the overall genetic variation in the population [17, 30].

There are ten identified pathogenic *Leptospira* species which can be further classified into more than 200 serovars currently known worldwide [42, 43]. Due to their extensive genetic diversity, pathogenic *Leptospira* can infect a wide array of hosts [1]. Many serovars, however, are commonly associated with a particular host reservoir [44]. A robust and accurate rapid typing method such as MLST can facilitate identification of *Leptospira* species and serovar, as well as possible infection clusters and transmission pathways. The application of simplified MLST scheme in this study demonstrated its potential use in infection surveillance and tracking of its possible source particularly during potential outbreaks. With the simplified scheme, analysis of clinical samples could be expedited and done directly without the need for culture, which present an advantage both in surveillance in routine samples and rapid identification of clusters and transmission pathways in outbreak settings. Targeting three markers with maximum TE and DP values equivalent to the full schemes also offers a cost-effective method with faster turnaround time of results since sequencing is also minimized. The method may also possibly detect unculturable *Leptospira* species which may reveal new STs or serovars. To date, there are 273 STs for scheme 2 recorded in the *Leptospira* PubMLST database [23]. This central database provides accurate ST assignment which can be used in clinical and epidemiologic studies. In the Philippines where leptospirosis is endemic and the number of cases continuously rises, it is imperative that a reliable rapid typing method be utilized in diagnostic laboratories to confirm clinically suspected cases and locate the source of infection. During past outbreaks, typing was done through culture, MAT, and other serological tests available which are indeed highly specific. However, because of the magnitude of outbreaks, results were confirmed several weeks after onset of symptoms [45, 46]. Such delay in release of test results not only did not aid in patient treatment but also in outbreak monitoring and management.

This study is the first to use MLST in human clinical specimens in the Philippines. Results of the simplified MLST scheme were consistent with results from MLST schemes 2 and 3. Although the samples were limited with only two different STs identified given the small number of samples evaluated, the study provides a first insight into clinical *Leptospira* species circulating in the Philippines. Study limitations are acknowledged. Firstly, because there is no a priori data on the prevalence of the serovars in the country and the limited available samples in the hospital, statistical requirements were not met when Philippine isolates alone were evaluated. Secondly, complete MLST profiles were not generated in all samples, hence the sequence type of the isolates could not be exactly assigned. Further investigations are still needed to confirm the suitability of the reduced scheme for disease surveillance. Serotype assignment of one isolate should also be validated, whether it may be assigned into a new ST. Also, since none of the full scheme alleles could be amplified, it is worth investigating if the inclusion of missing alleles may result in new STs. More samples should be tested in a longer period to assess all possible circulating strains in the country, and results should be verified by other standard tests like MAT. Different specimen types (blood, serum, urine, etc.) should be examined as well. With the difficulty in applying full MLST in clinical samples, this reduced scheme exhibits potential as a better strategy for outbreak and epidemiologic investigations. Although there are many platforms for *Leptospira* typing, the use of this simplified MLST scheme offers a rapid, accurate, and cost-effective method to identify, track, and monitor leptospirosis. Information from this study may be used as preliminary groundwork for more comprehensive epidemiologic studies on leptospirosis in the Philippines.

## Supporting information

S1 Fig Phylogeny of *L*. *interrogans* based on 16S rRNA (*rrs2*) gene sequence alignment (450 nt length). The ML tree was constructed with 1,000 replicates using K80 DNA substitution model. Clinical samples are in bold and their presumptive serovar and serogroup, and source location are shown. The tree was rooted with the outgroup, *L*. *kirschneri*. Bootstrap values inferred from ML and NJ are indicated in the nodes. Scale bar represents 0.0005 substitution per site.
(TIF)

S2 Fig Phylogeny of *L*. *interrogans* inferred from concatenated sequences of seven MLST loci (3243 nt length). Tree construction was based on TPM3uf+I+G DNA substitution model. Clinical samples are in bold. Serovar and serogroup assignment, and country of origin of samples of isolates are listed. The tree was rooted with *L*. *kirschneri*. Bootstrap values with 1,000 replicates inferred from NJ and ML are shown in the nodes. Scale bar represents 0.1 substitution per site.
(TIF)

S1 Table Genbank accession numbers of *Leptospira* in clinical samples. Submitted sequences range from 465 to 718 bp in length.
(DOCX)

S2 Table Primer sequences and annealing temperature used in PCR amplification of each gene target. Annealing temperatures are based on optimized PCR conditions for each gene target except for *glmU*, which was not amplified in all samples. ^a^Primers used for *Leptospira* detection. ^b^MLST schemes 2 and 3 primers published in *Leptospira* PubMLST database (http://pubmlst.org/leptospira/).
(DOCX)

S3 Table Reference isolates deposited in *Leptospira* PubMLST included in phylogenetic tree constructions. All information available on reference isolates in *Leptospira* PubMLST database are shown. STs are based on original MLST schemes. *Reference isolates with same STs associated with the isolates in the present study.
(DOCX)

S4 Table Allelic profiles of *Leptospira* in human clinical samples. Exact allele matches are shown for samples POM01, POM07, POM18, POM19, POM20 and POM23 partial profiles.—indicates no amplification or ambiguous sequences. *Nearest ST match to POM37 allelic profile. Numbers inside () show corresponding allele of nearest ST match to POM37.
(DOCX)

S5 Table Isolates deposited in *Leptospira* PubMLST with STs corresponding to the clinical samples in this study. (DOCX)
