# Supplementary figures and images for: Application of simplified MLST scheme for direct typing of clinical samples from human leptospirosis cases in a tertiary hospital in the Philippines
Source: PLoS One. 2021 Oct 20;16(10):e0258891. doi: 10.1371/journal.pone.0258891 (PMC8528318; doi:10.1371/journal.pone.0258891)

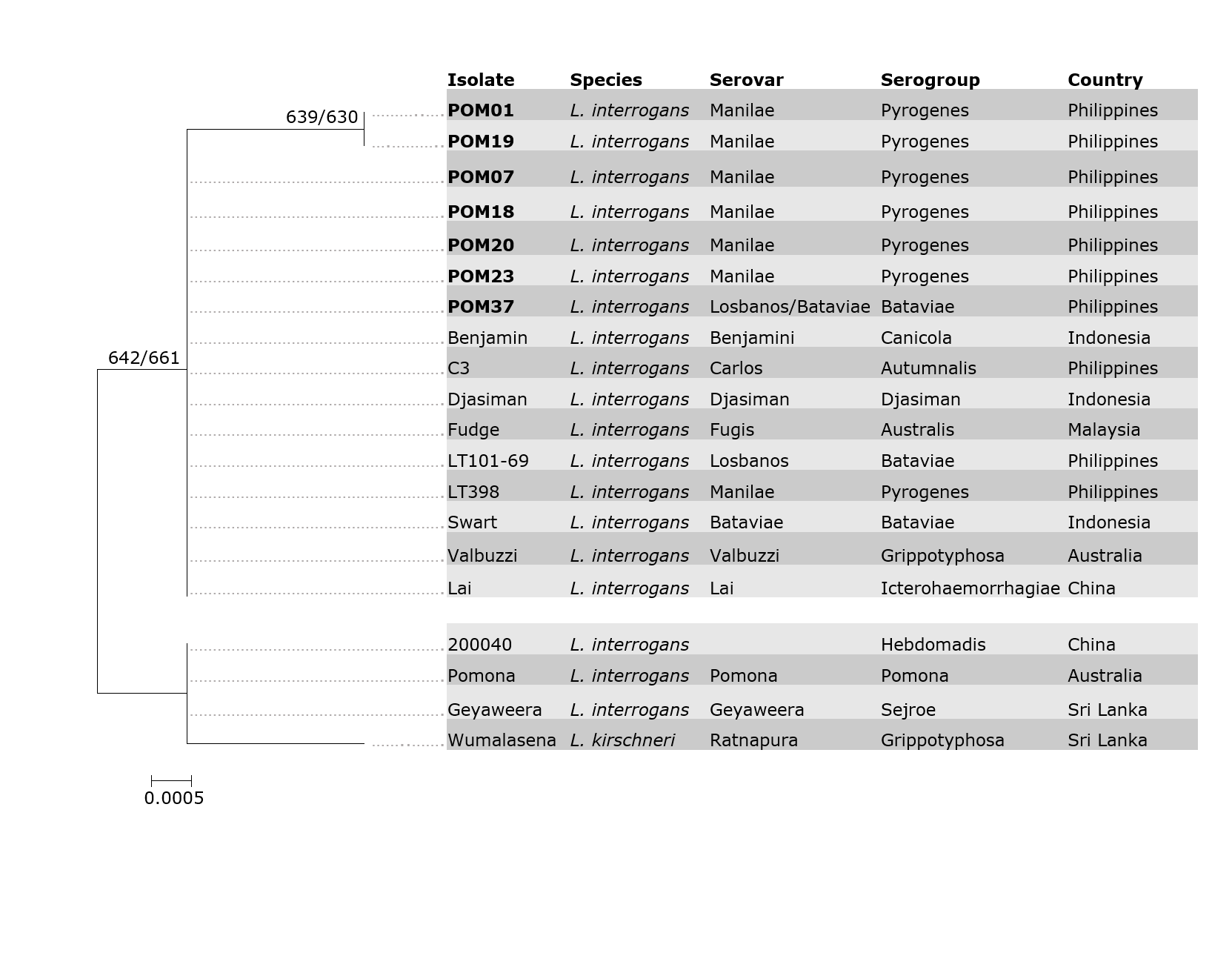

Supplement: S1 Fig — The ML tree was constructed with 1,000 replicates using K80 DNA substitution model. Clinical samples are in bold and their presumptive serovar and serogroup, and source location are shown. The tree was rooted with the outgroup, L. kirschneri. Bootstrap values inferred from ML and NJ are indicated in the nodes. Scale bar represents 0.0005 substitution per site. (TIF) [file pone.0258891.s001.tif]

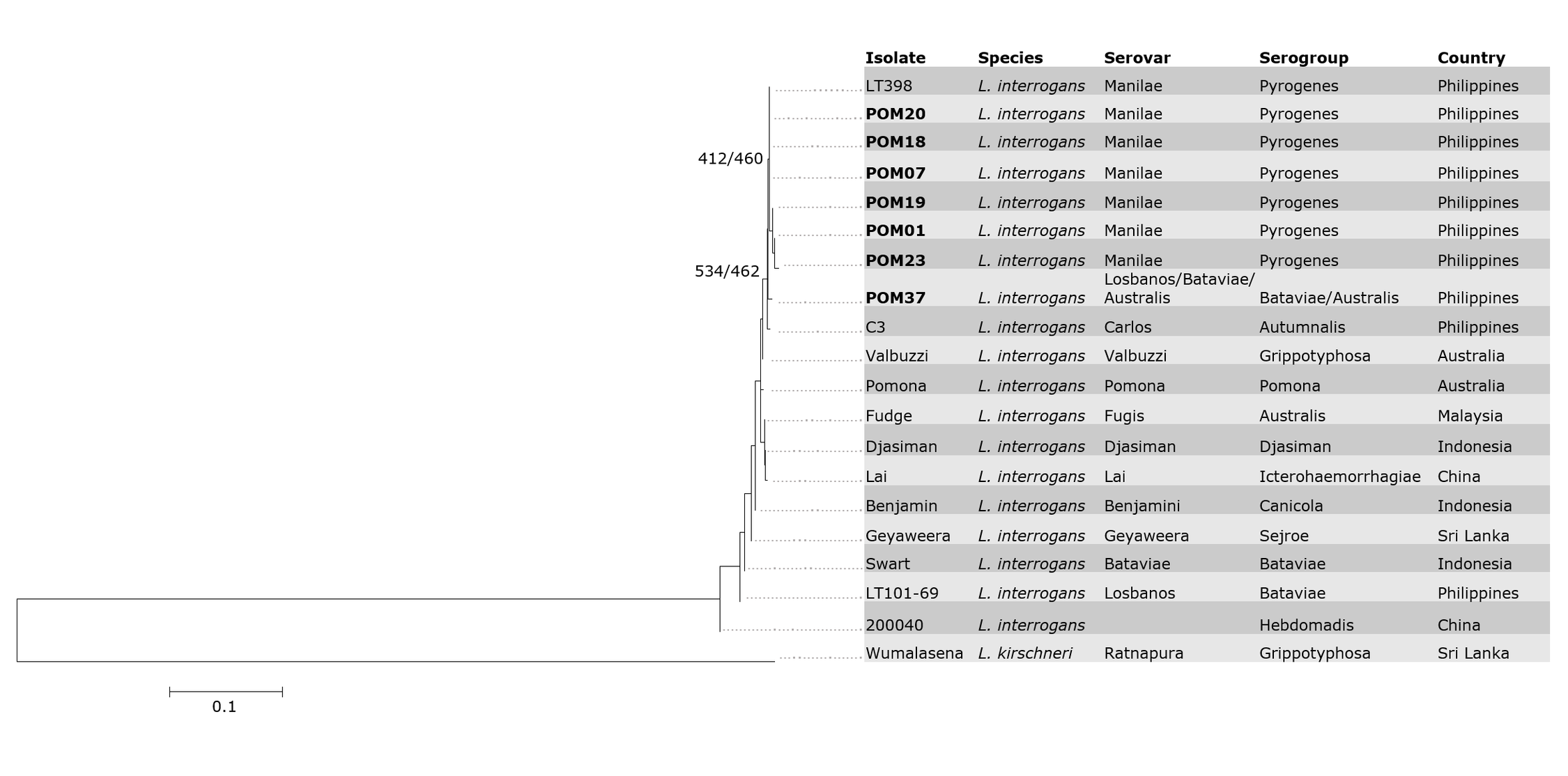

Supplement: S2 Fig — Tree construction was based on TPM3uf+I+G DNA substitution model. Clinical samples are in bold. Serovar and serogroup assignment, and country of origin of samples of isolates are listed. The tree was rooted with L. kirschneri. Bootstrap values with 1,000 replicates inferred from NJ and ML are shown in the nodes. Scale bar represents 0.1 substitution per site. (TIF) [file pone.0258891.s002.tif]
