# Supplementary material for: Application of simplified MLST scheme for direct typing of clinical samples from human leptospirosis cases in a tertiary hospital in the Philippines
Source: PLoS One. 2021 Oct 20;16(10):e0258891. doi: 10.1371/journal.pone.0258891 (PMC8528318; doi:10.1371/journal.pone.0258891)
Supplement: S1 Table — Submitted sequences range from 465 to 718 bp in length. (DOCX) [file pone.0258891.s003.docx]

**S1 Table. Genbank accession numbers of *Leptospira* in clinical samples.**

| **Sample**  **code** | ***16S/rrs2*** | ***lipL32*** | ***adk*** | ***lipL41*** | ***mreA*** | ***pntA*** | ***secY*** |
| --- | --- | --- | --- | --- | --- | --- | --- |
| POM01 | MT745827 | MT776334 | MT776355 | MT776341 | MT776362 | MT776348 | MT776369 |
| POM03 | MT745828 | - | - | - | - | - | - |
| POM07 | MT745829 | MT776335 | MT776356 | MT776342 | MT776363 | MT776349 | MT776370 |
| POM08 | MT745830 | - | - | - | - | - | - |
| POM18 | MT745831 | MT776336 | MT776357 | MT776343 | MT776364 | MT776350 | MT776371 |
| POM19 | MT745832 | MT776337 | MT776358 | MT776344 | MT776365 | MT776351 | MT776372 |
| POM20 | MT745833 | MT776338 | MT776359 | MT776345 | MT776366 | MT776352 | MT776373 |
| POM23 | MT745834 | MT776339 | MT776360 | MT776346 | MT776367 | MT776353 | MT776374 |
| POM37 | MT745835 | MT776340 | MT776361 | MT776347 | MT776368 | MT776354 | MT776375 |
