# Supplementary material for: Application of simplified MLST scheme for direct typing of clinical samples from human leptospirosis cases in a tertiary hospital in the Philippines
Source: PLoS One. 2021 Oct 20;16(10):e0258891. doi: 10.1371/journal.pone.0258891 (PMC8528318; doi:10.1371/journal.pone.0258891)
Supplement: S2 Table — Annealing temperatures are based on optimized PCR conditions for each gene target except for glmU, which was not amplified in all samples. aPrimers used for Leptospira detection. bMLST schemes 2 and 3 primers published in Leptospira PubMLST database (http://pubmlst.org/leptospira/). (DOCX) [file pone.0258891.s004.docx]

**S2 Table. Primer sequences and annealing temperature used in PCR amplification of each gene target.**

| **Gene** | **Primer Sequence (5' - 3')** | **Size (bp)** | **Annealing Temp** | **%GC content** | **Gene function/description** |
| --- | --- | --- | --- | --- | --- |
| 23S (*rrl*)^a^ | F:GACCCGAAGCCTGTCGAG  R:GCCATGCTTAGTCCCGATTAC | 482 | 54° | 67  52 | ribosomal RNA gene |
| 16S ribosomal RNA (*rrs2*)^a,b^ | F-CATGCAAGTCAAGCGGAGTA  R-AGTTGAGCCCGCAGTTTTC | 541 | 54° | 50  53 | ribosomal RNA gene |
| Lipoprotein L32 (*lipL32*)^a,b^ | F:ATCTCCGTTGCACTCTTTGC  R:ACCATCATCATCATCGTCCA | 474 | 56° | 50  45 | immunodominant outer membrane protein |
| Adenylate Kinase (*adk*)^b^ | F: GGGCTGGAAAAGGTACACAA  R: ACGCAAGCTCCTTTTGAATC | 531 | 56° | 50  45 | cellular energy homeostasis |
| Isocitarate dehydrogenase (*icdA*)^b^ | F: GGGACGAGATGACCAGGAT  R:CTTTTTTGAGATCCGCAGCTTT | 674 | 48° | 58  41 | isocitrate metabolic process |
| Lipoprotein L41 (*LipL41*)^b^ | F:TAGGAAATTGCGCAGCTACA  R:GCATCGAGAGGAATTAACATCA | 520 | 52° | 45  41 | outer membrane lipoprotein for cellular adhesion |
| UDP-N-acetylglucosamine pyrophosphorylase (*glmU*)^b^ | F:AGGATAAGGTCGCTGTGGTA  R:AGTTTTTTTCCGGAGTTTCT | 650 | - | 50  35 | transferase |
| NAD(P) transhydrogenase subunit alpha (*pntA*)^b^ | F:TAGGAAARATGAAACCRGGAAC R:AAGAAGCAAGATCCACAAYTAC | 621 | 52° | 40  38 | translocase (proton pump) |
| Rod shape-determining protein rodA (*mreA*)^b^ | F:GGCTCGCTCTYGACGGAAA  R:TCCRTAACTCATAAAMGACAAAGG | 719 | 52° | 61  36 | cell division and cell shape regulation |
| Pre-protein Translocase SecY subunit (*secY*)^b^ | F-ATGCCGATCATTTTTGCTTC  R-CCGTCCCTTAATTTTAGACTTCTTC | 549 | 56° | 40  40 | protein transmembrane transporter activity |
