# Supplementary material for: Application of simplified MLST scheme for direct typing of clinical samples from human leptospirosis cases in a tertiary hospital in the Philippines
Source: PLoS One. 2021 Oct 20;16(10):e0258891. doi: 10.1371/journal.pone.0258891 (PMC8528318; doi:10.1371/journal.pone.0258891)
Supplement: S3 Table — All information available on reference isolates in Leptospira PubMLST database are shown. STs are based on original MLST schemes. *Reference isolates with same STs associated with the isolates in the present study. (DOCX) [file pone.0258891.s005.docx]

**S3 Table. Reference isolates deposited in *Leptospira* PubMLST included in phylogenetic tree constructions.**

| **Id** | **Isolate** | **Country** | **Year** | **Host** | **Species** | **Serovar** | **Serogroup** | **Scheme 1** | **Scheme 2** | **Scheme 3** |
| --- | --- | --- | --- | --- | --- | --- | --- | --- | --- | --- |
|  |  |  |  |  |  |  |  | **ST** | **ST** | **ST** |
| 21 | Benjamin | Indonesia | 1937 | human | *L. interrogans* | Benjamini | Canicola | 16 | 27 | 68 |
| 24 | C3 | Philippines | 1970 | toad | *L. interrogans* | Carlos | Autumnalis | 60 | 19 | 25 |
| 30 | Djasiman | Indonesia | 1938 | human | *L. interrogans* | Djasiman | Djasiman | 11 | 32 | 77 |
| 33 | Fudge | Malaysia | 1957 | human | *L. interrogans* | Fugis | Australis | 137 | 14 | 19 |
| 35 | Geyaweera | Sri Lanka | 1965 | human | *L. interrogans* | Geyaweera | Sejroe | 44 | 43 | 83 |
| 44 | LT101-69* | Philippines |  | rat | *L. interrogans* | Losbanos | Bataviae | 50 | 25 | 44 |
| 45 | LT398* | Philippines | 1957 | rat | *L. interrogans* | Manilae | Pyrogenes | 57 | 12 | 9 |
| 57 | Pomona | Australia | 1936 | human | *L. interrogans* | Pomona | Pomona | 140 | 4 | 58 |
| 69 | Swart* | Indonesia | 1952 | human | *L. interrogans* | Bataviae | Bataviae | 50 | 24 | 44 |
| 71 | Valbuzzi | Australia | 1955 | human | *L. interrogans* | Valbuzzi | Grippotyphosa | 61 | 9 | 24 |
| 156 | Lai | China | 1958 | human | *L. interrogans* | Lai | Icterohaemorrhagiae | 1 |  | 47 |
| 1159 | 200040 | China | 2000 | human | *L. interrogans* |  | Hebdomadis | 89 | 240 | 155 |
| 101 | Wumalasena | Sri Lanka | 1965 | human | *L. kirschneri* | Ratnapura | Grippotyphosa | 116 | 95 | 92 |
