# Supplementary material for: Application of simplified MLST scheme for direct typing of clinical samples from human leptospirosis cases in a tertiary hospital in the Philippines
Source: PLoS One. 2021 Oct 20;16(10):e0258891. doi: 10.1371/journal.pone.0258891 (PMC8528318; doi:10.1371/journal.pone.0258891)
Supplement: S4 Table — Exact allele matches are shown for samples POM01, POM07, POM18, POM19, POM20 and POM23 partial profiles.—indicates no amplification or ambiguous sequences. *Nearest ST match to POM37 allelic profile. Numbers inside () show corresponding allele of nearest ST match to POM37. (DOCX) [file pone.0258891.s006.docx]

**S4 Table. Allelic profiles of *Leptospira* in human clinical samples.**

| **Sample**  **code** | **Simplified MLST Scheme^a^** | | | | **MLST Scheme 2** | | | | | | | | **MLST Scheme 3** | | | | | | |
| --- | --- | --- | --- | --- | --- | --- | --- | --- | --- | --- | --- | --- | --- | --- | --- | --- | --- | --- | --- |
|  | ***adk*** | ***lipL41*** | ***mreA*** | **ST** | ***adk*** | ***glmU*** | ***icdA*** | ***lipL32*** | ***lipL41*** | ***mreA*** | ***pntA*** | **ST** | ***adk*** | ***icdA*** | ***lipL32*** | ***lipL41*** | ***rrs2*** | ***secY*** | **ST** |
| POM01 | 5 | 8 | 8 | 12 | 5 | - | - | 2 | 8 | 8 | 6 | 12 | 1 | - | 2 | 5 | 1 | 8 | 9 |
| POM07 | 5 | 8 | 8 | 12 | 5 | - | - | 2 | 8 | 8 | 6 | 12 | 1 | - | 2 | 5 | 1 | 8 | 9 |
| POM18 | 5 | 8 | 8 | 12 | 5 | - | - | 2 | 8 | 8 | 6 | 12 | 1 | - | 2 | 5 | 1 | 8 | 9 |
| POM19 | 5 | 8 | 8 | 12 | 5 | - | - | 2 | 8 | 8 | 6 | 12 | 1 | - | 2 | 5 | 1 | 8 | 9 |
| POM20 | 5 | 8 | 8 | 12 | 5 | - | - | 2 | 8 | 8 | 6 | 12 | 1 | - | 2 | 5 | 1 | 8 | 9 |
| POM23 | 5 | 8 | 8 | 12 | 5 | - | - | 2 | 8 | 8 | 6 | 12 | 1 | - | 2 | 5 | 1 | 8 | 9 |
| POM37 | 3 | 8  (19,20) | 14 | 24,25* | 3 | - | - | 2 | 8  (19,20) | 14 | 6  (14,14) | 24,25* | 2 | - | 2 | 5  (8,2) | 1 | 5 | 44,49* |
