# Supplementary material for: Application of simplified MLST scheme for direct typing of clinical samples from human leptospirosis cases in a tertiary hospital in the Philippines
Source: PLoS One. 2021 Oct 20;16(10):e0258891. doi: 10.1371/journal.pone.0258891 (PMC8528318; doi:10.1371/journal.pone.0258891)
Supplement: S5 Table — (DOCX) [file pone.0258891.s007.docx]

**S5 Table. Isolates deposited in *Leptospira* PubMLST with STs corresponding to the clinical samples in this study.**

| **Id** | **Country** | **Year** | **Host** | **Isolate** | **Scheme 2** | **Scheme 3** | **Species** | **Serovar** | **Serogroup** |
| --- | --- | --- | --- | --- | --- | --- | --- | --- | --- |
|  |  |  |  |  | **ST** | **ST** |  |  |  |
| 45 | Philippines | 1957 | rat | LT398 | 12 | 9 | *L. interrogans* | Manilae | Pyrogenes |
| 945 | Unknown |  |  | Manilae str. K56 | 12 |  | *L. interrogans* | Manilae | Pyrogenes |
| 951 | Unknown |  |  | Manilae str. UP-OM | 12 |  | *L. interrogans* | Manilae | Pyrogenes |
| 1214 | Japan | 2012 | mouse | UP-MMC-NIID HP | 12 | 9 | *L. interrogans* | Manilae | Pyrogenes |
| 1215 | Japan | 2012 | mouse | UP-MMC-NIID LP | 12 | 9 | *L. interrogans* | Manilae | Pyrogenes |
| 69 | Indonesia | 1952 | human | Swart | 24 | 44 | *L. interrogans* | Bataviae | Bataviae |
| 44 | Philippines |  | rat | LT101-69 | 25 | 44 | *L. interrogans* | Losbanos | Bataviae |
| 19 | Australia | 1934 | human | Ballico | 13 | 49 | *L. interrogans* | Australis | Australis |
| 809 | Unknown |  |  | SRR507773 |  | 49 | *L. interrogans* |  |  |
| 877 | Unknown |  |  | Australis str. 200703203 | 98 | 49,153 | *L. interrogans* |  |  |
| 1225 | Unknown |  |  | SU5 |  | 49 | *L. interrogans* |  |  |
